# Cancer patients’ perspectives on remote monitoring at home during the COVID-19 pandemic- a qualitative study in Norway

**DOI:** 10.1186/s12913-022-07897-4

**Published:** 2022-04-06

**Authors:** Ann-Chatrin Linqvist Leonardsen, Ann Karin Helgesen, Andreas Stensvold, Jannik Magnussen, Vigdis A. Grøndahl

**Affiliations:** 1 Department of Health, Welfare and Organization, Østfold University College/Østfold Hospital Trust, Postal box code (PB) 700, 1757, Halden, Norway; 2 Department of Health, Welfare and Organization, Østfold University College, PB 700, NO-1757, Halden, Norway; 3 Cancer Department, Østfold Hospital Trust, Postal box code 300, NO-1714, Grålum, Norway; 4 Norwegian Cancer Society, Østfold, Norway

**Keywords:** Remote monitoring, Cancer care, COVID-19, Patients’ experiences

## Abstract

**Background:**

The COVID-19 pandemic triggered an unprecedented demand for digital health technology solutions, such as remote monitoring. Previous research has focused on patients with chronic diseases, and their experiences with remote monitoring during the pandemic. Several recommendations have been presented to reduce the frequency of cancer patients’ visits to oncology centers and minimizing the risk of exposure to COVID-19, such as remote monitoring. However, few studies have explored how this has influenced the healthcare services to cancer patients.

**Aim:**

To explore cancer patients’ perspectives on remote monitoring at home during the COVID-19 pandemic.

**Design:**

The study had a qualitative design, using in-depth, individual interviews.

**Methods:**

A total of eleven interviews were conducted with patients who received remote monitoring during the COVID-19 outbreak. Three of the interviews were conducted by telephone, and eight on a digital platform, audio recorded, and transcribed verbatime. Data were analyzed using reflexive thematic analysis as recommended by Braun & Clarke.

**Results:**

All participants were conscious about being vulnerable to infections due to having cancer and receiving cancer treatment, and the pandemic to them represented an extra burden. Most of the participants experienced that their healthcare services had changed due to the pandemic, but there was no consensus on how the services had changed. All of the participants presented remote monitoring as something «new». Whether they received remote monitoring by telephone, video consultations or more advanced solutions with the possibility to complete a questionnaire or fill in measurements, did not seem to impact their views. However, all agreed that remote monitoring could never totally replace physical consultations in hospital. Participants’ views seemed to grow more positive over time, but still they emphasized both positive and negative aspects of remote monitoring solutions in cancer care.

**Conclusion:**

Remote monitoring was introduced as a necessity in cancer care during the COVID-19 outbreak. This may seem as an efficient solution, allowing for patients to stay at home and avoid infection. Our results indicate that, in the case of cancer patients, it is important that healthcare personnel balance the remote monitoring solution with person-to-person contact.

## Background

The declaration of the COVID-19 pandemic lead to an extreme pressure on already pressured healthcare services worldwide [1, 2]. Even before the pandemic, healthcare services were facing extensive challenges due to the increased proportion of elderly persons, and persons with long-lasting disease [3, 4]. In addition, there were not enough healthcare professionals to manage the increasingly complex patient care needs within an increasingly complex healthcare system [5]. Consequently, the COVID-19 pandemic forced healthcare services to implement digital solutions for patient management and monitoring as a replacement of person-to-person meetings. The World Health Organization (WHO) declared that “This pandemic has triggered an unprecedented demand for digital health technology solutions and has revealed successful solutions such as for population screening, tracking the infection, prioritizing the use and allocation of resources, and designing targeted responses” [6].

Technology enabled care (TEC) is a collective term for telecare, telehealth, telemedicine, mHealth, self-care apps, digital health and e-health services [7]. TEC has increasingly been seen as an integral part of the solution to many of the challenges facing the health sector. This has also been the case in solutions for remote patient monitoring, where patients outside conventional clinical settings have been monitored with help of technology, with the aims of increasing access to care and decrease healthcare delivery costs [8, 9]. When delivering TEC, interactions can either be synchronous (occurring in real time), asynchronous (not occurring in real time), or a combination of both [10].

A 2021 systematic review of research focusing on remote monitoring systems in primary healthcare [11], found that studies focused mostly on patients with diabetes and cardiovascular diseases. Moreover, during the evaluation of the implementation of these interventions, the major difficulty was the integration of information into already existing systems in the primary healthcare infrastructure and in changing working processes of primary healthcare professionals. Another 2021 systematic review of reviews [12] also included studies mainly focusing on patients with diabetes or hypertension. The authors concluded that remote monitoring facilitates data transmission, analysis, and feedback, but does not necessarily assist patients in making or sustaining medication, diet, or physical activity change that are often necessary for chronic conditions. Moreover, the authors claim that remote monitoring could be seen as a bridge to necessary further support, but not superior by itself to usual care. A 2020 review analyzing the implementation and impact of remote home monitoring models (virtual wards) during COVID-19 [13] concluded that future research should focus on staff and patient experiences of care and inequalities in patients’ access to care related to remote monitoring.

Patients with cancer are particularly vulnerable to the SARS-CoV-2 virus and require clinical vigilance to prevent infection [14, 15]. Several recommendations have been presented to reduce the frequency of cancer patients’ visits to oncology centers while maintaining access to cancer therapy and minimizing the risk of exposure to COVID-19, such as activating telemedicine and applying innovative ideas to minimize patients’ visits in hospital [16]. A recent study explored cancer patients’ perspectives on remote patient monitoring for COVID-19 [17]. Results indicated that patients appreciated that the remote patient monitoring provided a clinical safety net and a link to their clinical team during a period of isolation. Moreover, patients appreciated that remote patient monitoring provided education on the virus and symptom management. Research on remote monitoring of patients with lung cancer indicated that they felt well informed, but that they lacked preparation for the full extent of the problems they experienced [18]. Another study found that when daily automated monitoring, self-management coaching and follow-ups using guideline-based decision support were combined with in-between-visit care, there were significant reductions in symptom burden overall for cancer patients beginning chemotherapy [19]. Barsom et al. (2020) found that videoconference was equal to face-to face consultation in terms of patient satisfaction and perceived quality of care [20]. However, we have found few studies exploring cancer patients’ experiences with remote monitoring from hospital at home during the COVID-19 outbreak. Consequently, the aim of the study presented here was to explore cancer patients’ perspectives on remote monitoring at home during the COVID-19 pandemic.

## Materials and methods

The study had a qualitative design, using in-depth, individual interviews to explore patients’ perspectives on remote monitoring during the COVID-19 pandemic. A qualitative approach is appropriate when aiming to explore how individuals experience a phenomenon, dependent on their background, interests and interpretation [21]. The study is in-line with the Consolidated criteria for reporting qualitative research – COREQ [22].

### Setting and sample

The study was conducted in a hospital with a catchment area of approximately 320.000 inhabitants. The hospitals’ healthcare services for cancer patients are divided in an in-patient ward and an out-patient clinic. Healthcare personnel are primarily employed both places, and include oncologists, registered nurses and nurses specialized in cancer care. The technology explored in this study was introduced in 2016, and applied in order to limit the need for patients to attend to the hospital. The technology includes solutions for remote monitoring of patients through either mobile cell phones, video conferences, or software solutions included in tablets or computers, also allowing for patients to provide feed-back through responding to questionnaires on a daily basis.

We used a purposive sampling method, selecting information rich cases for in-depth study, from the patient group that is remotely monitored. Maximum variation sampling was sought [23], focusing on inviting patients of both genders, a range in age, with different socio-economical background and from different geographical areas (central/rural). Inclusion criteria were patients over 18 years of age, able to give a written consent to participate, able to understand and express themselves in Norwegian, and have experienced remote monitoring in the period from March 12th (the COVID-19 outbreak) to the date of interview. Patients fulfilling the inclusion criteria were invited to participate by a study nurse working in the cancer department, which was not part of the research team. After having consented to participate, the patients’ contact information was shared with the researchers, who then made the interview agreement.

### Interview guide

A semi-structured interview guide was developed based on previous research on cancer patients’ experiences with remote monitoring e.g. [17, 18], and through several discussions within the research group until consensus was reached (see Table 1).

**Table 1:** Interview guide

1. Can you please tell me a little about your health, and why you are being remotely monitored by the cancer ward?	
2. Can you please describe the remote monitoring solution you use? Follow up: Do this require any extra equipment?	
3. Is there any equipment you are more or less comfortable with? Follow up: Can you please elaborate on that?	
4. Did you get any information or training before starting with the remote monitoring solution? Follow up: Can you please describe this further?	
5. Can you describe a day which includes remote monitoring?	
What is important to you when meeting the cancer ward personnel?	
6. Can you describe your need for contact with a nurse? Follow ups: What do you need? Why do you need this? Is your need covered?	
8. Can you describe your need for contact with a physician? Follow ups: What do you need? Why do you need this? Is your need covered?	
9. Did something change in your health service after the presentation of COVID-19? Please elaborate.	
10. Do you feel you have an impact on the services you receive?	
11. Is there something especially positive about remote monitoring?	
12. Is there something especially negative about remote monitoring?	
13. Is there something you want to tell me about regarding your services during COVID-19 that I have not asked you about?	

The research group consists of three registered nurses (female) with extensive clinical experience as well as experience with qualitative research methods (ACLL, AKH, VAG), two oncologists (one of them AS, both male), one registered nurse (female) working in the cancer department and one user representative (JM, female). In the current study, only five of the research group members took part in analysis and writing of the paper, due to confidentiality issues.

### Data collection

After having consented to participate, patients were contacted by one of the researchers, and an appointment for an interview was set. Due to the COVID-19 pandemic, interviews needed to be conducted remotely, preferably through the safe digital platform Zoom. Participants who did not desire to participate digitally, were offered to participate per telephone. Participants received an email describing how to open the platform. In total three patients were interviewed by telephone, the rest through Zoom (ACLL, AKH or VAG). Both the interviews conducted in Zoom and the telephone interviews provided rich data for analysis.

Before and after each interview the researchers wrote down thoughts and impressions that could possible impact the interview, or the interpretation of what was said, as a method of reflexivity [24]. Interviews (sound only) were digitally recorded, and transcribed verbatim by an external transcriber, who had signed a non-disclosure agreement. The interviews lasted from 20 to 44 min (median 35 min).

### Analysis

To analyze the data we used a reflexive, thematic analysis according to recommendations from Braun & Clarke [24, 25]. The analysis consisted of four steps. In step one, the first author read and re-read the transcripts to get an impression of the whole, and to familiarize with the data. Then, each transcript respectively was coded inductively, by manually marking central key words/concepts that could represent a code (ACL). In step two, codes across all interviews were collated, and the first author searched for concepts that were similar or differed, and that could be identified as sub-themes across all interviews. The codes and sub-themes were then, in step three, assessed and discussed by three of the authors (ACLL, AKH and VAG), all experienced with qualitative research studies, until consensus was reached. Further, in step four, the sub-themes were reviewed, also including impressions and pre-conceptions from the reflexivity notes, and the final themes and sub-themes were identified through iterative discussions among the three authors until consensus was reached. Then, all authors approved on the final results. Table 2 presents an example of the analysis process.

**Table 2:** Example of the analysis process

Transcript	Codes (step 1)	Sub-theme (step 2-3)	Theme (step 4)
P 4: … when I read it, I thought that it may be a bad solution and not enough for what I wished for … want to be examined …. But, after I had spoken to her, I think we spoke for 45 min, had good time, then I felt more safe, and got an overview of future consultations. Now, I have been under treament for over one year, and you get used to thing … But, sometimes, you need the re-assurance that things will work out fine, you get the services you need … But, we are kind of more alone in this … .	Bad solution Not enough Want to be examined Had good time Got an overview Get used to Need re-assurance Get what you need More alone	Ambivalent perspectives	Remote monitoring can not replace human contact

*P* participant

## Results

In total 11 in-depth, individual interviews were conducted in the period March to May 2021. Six of the participants were female, five were male, their mean age was 56 years (range 45-83). Four of the participants received remote monitoring through telephone calls only, while five participants had used video-conference through Skype or Zoom. Only two of the participants had used a digital solution which included answering questions about their condition on a daily basis, which were submitted on-line, allowing for healthcare personnel to assess the response and respond if needed.

Through analysis we identified two main themes with related subthemes; 1) The pandemic-an extra burden, with subthemes a) views of physical hospital visits, and b) changed services, and 2) Remote monitoring cannot replace human contact, with subthemes a) ambivalent perspectives, b) technological challenges, and c) saves time and energy. Results are supported by illustrative quotes, marked with participant number (participant = P) in parenthesis.

### The pandemic-an extra burden

All of the participants were conscious about being vulnerable to infections due to having cancer and receiving cancer treatment. Hence, the pandemic to them represented an extra burden. Their views on physical visits to the hospital differed. Moreover, most of the participants experienced that their healthcare services had changed due to the pandemic, but there was no consensus on how the services had changed.

#### Views of physical hospital visits

All of the participants stated that even if they had received remote monitoring services during the pandemic, they still needed frequent physical hospital visits for medical treatment such as chemotherapy. Their views on attending the hospital was two-folded: some perceived that physical visits to the hospital were an increased risk for getting COVID-19 or any other infection. For example, participant 6 stated:

«*If we try to avoid this (the virus) … the hospital isn’t the right place to be. I wasn’t very interested in going to the hospital, staying in the corridors there»*

Others perceived that the hospital was safer than other public areas due to the heavy limitations. This indicates that participants were aware of the risk for catching illness when in public, and that this was something they reflected about. Participant 1 described that the situation regarding risk of catching the virus due to contact with other people had decreased due to everybody using face masks. Participant 11 stated:

«*Early during corona, it was no control at all, I think. But, afterwards this has become our safety, because the initiatives have led to the hospital feeling safer»*

About half of the participants mentioned that they felt that the hospital was «the safest place to be» due to strict legislations, and since the frequent physical visits were necessary they appreciated this. Participant 9 quoted:

«*I’m not so stressed, I do not get near other people anyway … Life is too short to worry, I rather feel taken care of … so, I’m not afraid to go to the hospital”.*

Hence, the participants’ perspectives on whether it was safe or not in hospital for them as being sick to cancer and with an increased risk of getting the COVID-19 virus varied. This did not seem to relate to whether the participant was in a terminal phase of the disease or not.

#### Changed services

All of the participants experienced that the healthcare services had changed due to COVID-19. But, the way the services had changed varied, according the participants. All of the participants experienced that physical meetings were replaced by remote monitoring. Still, some also stated that they experienced having a choice regarding remote monitoring. For example, participant 7 prompted:

«*If I don’t want the remote monitoring it’s no problem. I often get a choice … But, I think that if it wasn’t for the corona, remote monitoring wouldn’t have been an issue … No questions about that before the pandemic … But, I think that remote monitoring has been positive»*

Some of the participants emphasized feeling that the remote services had limited their services too much. E.g. participant 5 reported:

« *… getting treatment trough physical meetings versus always feeling alone is the worst...and all the follow-ups in-between chemo were cancelled … So, I feel that this has been tougher … ”*

This was supported by e.g. participant 4, who experienced lacking services that were offered in «non-pandemic» times, which she had read about in information brochures:

« *… the negative is that you feel … that the examination should have been physical, and was not … The conversation is okay, but she (the physician) said that she was meant to examine my breasts, and I got a remote monitoring appointment»*

Due to the cancer diagnosis, several of the participants reported being vulnerable, and needing lots of information about the progress of their illness, about the treatment and about adverse-effects of the treatment. In this situation, many of the participants emphasized the importance of including relatives in both remote and physical consultations. Participant 4 had experienced that relatives were not allowed in the hospital, even when receiving information about the diagnosis the first time:

« *… one could wish, and it is also recommended in hospital, as she (the physician) told me at my first control after I got the cancer diagnosis, that I should bring along a relative because four ears hear better than two … But, they (the relatives) were stopped at the entrance … »*

Regarding relatives attending the hospital visits, this varied between participants. Participant 5 prompted:

«*..you don’t always catch what’s been said.. It depends on your own mental condition. And they often recommend to bring a person. I took my husband to the consultations, and he heard other things than I did, because I was black, but he also heard the positive feed-back»*

Hence, there was no agreement across participants on whether the remote monitoring solution represented equal health service as “traditional health services” to cancer patients- or if the “change” was to the better or to the worse. It seemed like participants who had experienced missing information or missing out on services they expected to receive, were the most negative to remote monitoring replacing physical consultations.

### Remote monitoring cannot replace human contact

All of the participants presented remote monitoring as something «new», and their views on this solution varied. Whether they received remote monitoring by telephone, video consultations or more advanced solutions with the possibility to complete a questionnaire or fill in measurements, did not seem to impact their views. However, all agreed that remote monitoring could never totally replace physical consultations in hospital. Participants’ views seemed to grow more positive over time, but still they emphasized both positive and negative aspects of remote monitoring solutions in cancer care.

#### Ambivalent perspectives

Most of the participants reported initially feeling uncertain about remote monitoring. This was often due to participants not feeling familiar with video consultations. Participant 9 elaborated:

«*When you have a severe illness, there’s something about meeting a person because you are uncertain about so many things … When sitting like we do now (video interview), after having hung-up, because you are anxious about results and you forget to ask, even if you have written it down … The focus on the screen takes over, I catch what she (the physician) says, but then I forget. For my next appointment I have asked to come to the hospital»*

Moreover, several of the participants felt that remote monitoring represented a kind of distance between them and the physician or nurse. Participant 5 stated:

«*The limit for talking about things gets higher during video consultations. You think that the time is limited*, *and then you try to just say what’s absolutely needed … Body language is absent, and short anecdotes or comments as well, gets somewhat more formal … The little details that gives a lot of information are gone»*

Still, all of the participants, except one, reported that remote monitoring filled its’ mission allowing for follow-ups in-between physical visits to hospital due to needing treatment or physical examinations. However, several of the participants described that remote monitoring had to be initiated after having met the physicians and nurses in person. Participant 11 described it like this:

«*It’s human-to-human meetings in physical presence, sitting on a chair beside the physicians’ desk … You get another sort of contact, eye, body, everything … I think this takes longer time through remote monitoring … I think the first meetings should be face-to-face … »*

Participants who initially felt uncertain about remote monitoring reported that this changed after having used this some time. Participant 4 explained:

«*When I read about it, I thought it might be a bad solution, not good enough … But, after we talked for about 45 minutes, I felt more safe … »*

The participants appreciated that physicians indicated having much time available during the remote monitoring session, in contrast to feeling «in-line» at the hospital. Moreover, remote monitoring easily allowed for participants to get in touch with healthcare personnel when needed. Participant 1 prompted:

«*I know I can call if there’s something I have forgotten … Not as easy if you’ve been to a physician, overloaded with information and walks out the door. Then you’re finished … It’s easier to be followed-up, and after a phone call it’s just for me to call back if needed.»*

Participants were on one hand satisfied with avoiding the long travel distance to hospital, but on the other hand they were critical to remote monitoring totally replacing physical consultations. In addition, participants’ reports underline the importance of proper information and support when being offered remote monitoring.

#### Technological challenges

Several of the participants reported to be familiar with technology and digital meetings before the pandemic, even if not in relation to their illness. They perceived that having this competence in front was an advantage, and some reflected about how remote monitoring could feel like for patients without such competence. Regardless of this, all of the participants emphasized the importance of avoiding technological challenges, which several of them had experienced. Participant 9 quoted:

«*It’s quite bad..lagging, and she talks and I hear it afterwards … Last time the net fell out..»*

Participant 5 also stated that the remote monitoring solution required updated equipment at home. She elaborated;

«*If the connection is bad, and the systems are lagging so that we cannot talk as we want, it gets very negative. In the beginning, it went black, lagged … I had to improve my system at home, and I think the hospital did some adjustments as well. It requires a proper internet, and fast equipment»*

Participant 11 told about being without internet coverage, having to wait in bad weather, and having to update or re-install applications. He stated:

«*It has to be user-adjusted. It requires that you understand the user interface, dare to download the app, make updates and so on … »*

These findings underline the importance of patient education when using remote monitoring, and to ensure that patients have proper equipment for such services at home.

#### Saves time and energy

Most of the participants appreciated that remote monitoring was effective, and saved time. Several of the participants reported of periods with feeling exhausted due to deterioration or treatment side-effects. Then, not having to go to the hospital, waiting in line in a crowded waiting-room, and spending two-three hours in hospital felt like a relief. Participant 3 said:

«*You can do it at home, doesn’t take more than half an hour … Otherwise, you have to go back and forth, take blood-samples, then a long time before the treatment, and then I get two days at least feeling exhausted … »*

This was supported by most of the participants. Participant 1 also emphasized other positive effects of remote monitoring:

«*Even if the physician or nurse is delayed, it doesn’t matter. Everything takes less time. Just have to be ready for a call in two minutes, I have it with me (the cell phone), it’s the same whether it is now or in ten minutes»*

In addition, several of the participants felt like remote monitoring allowed them to «live like normal», not having to adjust their lives to «being sick». Participant 5 stated:

«*With remote monitoring I can be at work. Just plan for a meeting, like any other meeting … And from home-office as well, just log-off and log-on»*

Participant 8 also elaborated:

« … *and I don’t even have to be at home. I can sit in my caravan in the mountains and make the appointment there*»

Hence, remote monitoring did not just save energy and time, but also allowed participants to use their energy in «good periods» on meaningful activities. Cancer patients are critically ill, as were the participants in this study. The findings in this study indicate that it is important for them to choose for themselves what they spend their valuable (and sometimes limited) time on.

## Discussion

We found that patients experienced the COVID-19 pandemic as an extra burden in addition to having a cancer diagnosis. Hospital visits were both perceived as safer than other out-of-home activities, and as a risk. All of the participants had experienced that services changed due to the pandemic. Moreover, they agreed that remote monitoring never could totally replace physical meetings. Nevertheless, they appreciated some advantages, such as remote monitoring saving time and energy.

Participants in our study reported that services had changed due to the COVID-19 pandemic. Some experienced more limited services, while others experienced a more frequent contact with healthcare personnel due to the remote monitoring solution. A 2021 systematic review identified a reduction in routine activity of cancer services, such as a delay in surgeries or radiotherapy, as well as rescheduling or cancellation of outpatient visits [26]. Cancer patients are in an extremely vulnerable situation, having to deal with complex medical information, make difficult medical decisions, cope with an uncertain prognosis and radical treatments, with sometimes limited guarantees for improvement [27, 28]. The COVID-19 pandemic adds to this, since patients with cancer appear to be more vulnerable to worse outcomes from the infection, including greater need for ventilator support [14] and elevated mortality rates [29]. Lou et al. [30] found that patients undergoing active treatment for cancer were most concerned about the short-term effects of the COVID-19 pandemic on the logistics as well as potential efficacy of ongoing cancer treatment. In addition, cancer patients were concerned that the population at large did not see the health implications of COVID-19. Some of the patients in our study experienced physical appointments in hospital as an increased risk, while others perceived that the hospital was «safer than ever» due to the many restrictions following the pandemic. Cancer patients with different diagnoses and prognoses require different channels and contents of communication from their providers [31]. Our findings show that this is also relevant in the situation of a pandemic.

All of the participants in our study agreed that remote monitoring could never totally replace person-to-person meetings. Even if participants’ views seemed to grow more positive over time, they emphasized both positive and negative effects of the remote monitoring. Studies have shown that cancer patients appreciate that remote monitoring provides information and a clinical safety net, also being a link to the nurses and physicians during a period of isolation [17, 32, 33]. Technological barriers, such as software defects, access to video-compatible devices, access to high-speed internet, and individual technological fluency has been associated with decreased satisfaction [34, 35]. This was also discovered in our study.

Interestingly, several of our participants were monitored through telephone, and three of the patients chose to conduct telephone interviews. Similarly, a study of 385 cancer patients found that the preferred method for communication was a phone call with a 92% response rate followed by the electronic patient portal, mobile application, telemedicine and text message in 75, 76, 73, and 72%, respectively, all as a substitute to in-person interaction with their physicians [36]. Even if research on video consultations is sparse, some studies indicate that such consultations offer potential advantages to patients (who are spared the cost and inconvenience of travel) and the healthcare system (eg, they may be more cost-effective) [37, 38]. This was also supported by results in our study.

Cancer care often involves a team of health professionals, which necessitates complex interconnected communications for optimal clinical decision making [39]. It has been claimed that health care providers find it challenging to discuss life-threatening health conditions with their patients, including cancer-related communication [40]. Margolin et al. [35] found that 78% of patients and 85% of physicians “strongly agreed” that they were able to discuss sensitive topics about cancer care remotely as well as they could at an in-person visit. This emphasized the need to develop and implement more evidence-based interventions to engage patients, enhance patient-provider communication, and facilitate shared decision making to improve patient-centered health outcomes [41].

### Strengths and limitations

Conducting interviews through a digital platform or by telephone may have led to us not being able to assess participants’ expressions, emotions and non-verbal alterations. However, in-person interviews are assumed to be only slightly superior to video calls [42], and due to the pandemic we had no other alternative. In addition, the sample was quite small, and the technology used was limited. Still, the interviews provided rich data, also supported by previous studies on remote monitoring of cancer patients, which increases the transferability and validity of our findings. The research group consisted of both experienced nurses, oncologists and a user representative. Moreover, we used reflexivity as a method, focusing on limiting the impact of our own assumptions. This increases the trustworthiness of our results.

## Conclusion

The COVID-19 pandemic added an extra challenge for cancer patients, due to already being vulnerable. Physical consultations were replaced with remote monitoring solutions, which represented both advantages and disadvantages.

### Implications for clinical practice

Our findings indicate that cancer patients are not a homogeneous group of people, but have to be seen as individuals. In the future it is important for healthcare personnel to focus on limiting the disadvantages, e.g. through finding viable solutions that do not require specific equipment, skills or a fast internet-connection. In addition, it is essential that remote monitoring do not totally replace the person-to-person meetings, suggesting that the initial meetings are physical to build a relationship between patient and personnel.

## Data Availability

Datasets generated and/or analysed during the current study are not publicly available due to local ownership of data, but aggregated data are available from the corresponding author on reasonable request.
